# Calibration strategies for use of the nanoDot OSLD in CT applications

**DOI:** 10.1002/acm2.12491

**Published:** 2018-11-13

**Authors:** Sarah B. Scarboro, Dianna Cody, Francesco C. Stingo, Paola Alvarez, David Followill, Laurence Court, Di Zhang, Michael McNitt‐Gray, Stephen F. Kry

**Affiliations:** ^1^ The University of Texas MD Anderson Cancer Center Houston TX USA; ^2^ Graduate School of Biomedical Sciences The University of Texas Health Science Center Houston Houston TX USA; ^3^ Biomedical Physics Graduate Program David Geffen School of Medicine at UCLA Los Angeles CA USA; ^4^ The Department of Radiological Sciences David Geffen School of Medicine at UCLA Los Angeles CA USA; ^5^ Present address: Toshiba American Medical Systems Tustin CA USA

**Keywords:** calibration, CT, dosimetry, OSLD

## Abstract

Aluminum oxide based optically stimulated luminescent dosimeters (OSLD) have been recognized as a useful dosimeter for measuring CT dose, particularly for patient dose measurements. Despite the increasing use of this dosimeter, appropriate dosimeter calibration techniques have not been established in the literature; while the manufacturer offers a calibration procedure, it is known to have relatively large uncertainties. The purpose of this work was to evaluate two clinical approaches for calibrating these dosimeters for CT applications, and to determine the uncertainty associated with measurements using these techniques. Three unique calibration procedures were used to calculate dose for a range of CT conditions using a commercially available OSLD and reader. The three calibration procedures included calibration (a) using the vendor‐provided method, (b) relative to a 120 kVp CT spectrum in air, and (c) relative to a megavoltage beam (implemented with ^60^Co). The dose measured using each of these approaches was compared to dose measured using a calibrated farmer‐type ion chamber. Finally, the uncertainty in the dose measured using each approach was determined. For the CT and megavoltage calibration methods, the dose measured using the OSLD nanoDot was within 5% of the dose measured using an ion chamber for a wide range of different CT scan parameters (80–140 kVp, and with measurements at a range of positions). When calibrated using the vendor‐recommended protocol, the OSLD measured doses were on average 15.5% lower than ion chamber doses. Two clinical calibration techniques have been evaluated and are presented in this work as alternatives to the vendor‐provided calibration approach. These techniques provide high precision for OSLD‐based measurements in a CT environment.

## 1 Introduction

The nanoDot optically stimulated luminescent dosimeter (OSLD) (Landauer Inc, Glenwood, IL, USA), is a common detector for point dosimetry, particularly in therapeutic applications.^1^, ^2^, ^3^, ^4^, ^5^, ^6^, ^7^ More recently, in conjunction with the increased interest in improved computed tomography (CT) dosimetry, there has been interest in using the nanoDot for this purpose. Several recent studies have used OSLD in this capacity,^8^, ^9^, ^10^, ^11^, ^12^, ^13^, ^14^ including a few in‐depth evaluations of the dosimeter characteristics in a CT environment.^15^, ^16^, ^17^, ^18^ Importantly, no studies to date explore or describe calibration procedures for OSL‐based dosimetry in a CT environment. This is not just practically useful information when conducting these measurements, but is particularly relevant in the context of dosimetric uncertainty. While the AAPM TG‐191 report^19^ details dosimetric uncertainty associated with more common applications, the uncertainty of OSLD measurements in CT dosimetry has not yet been quantified.

The general formalism for calculating dose using this dosimeter is shown in eq. (1),^19^ where the absorbed dose, *D*, at the location of the OSLD is equal to product of the average corrected signal reading (*M*_*corr*_), the calibration coefficient (*N*_*D,W*_), and any additional necessary correction factors (*k*).

(1) $$D={\bar{M}}_{\mathrm{corr}}\times N_{D,W}\times k_{L}\times k_{F}\times k_{G}\times k_{\theta }\times k_{Q}$$

The average corrected signal, ${\bar{M}}_{\mathrm{corr}}$, accounts for signal depletion (*k*_*d*_; determined during commissioning), the number of readings of the detector (J), background if necessary (*M*_*bkg*_), and, if a batch calibration approach is used, the unique element sensitivity (*k*_*s,i*_), as shown in eq. (2):

(2) $$M_{\mathrm{corr}}=k_{s,i}\cdot \left[\frac{\sum_{j}\left(M_{raw,j,i}\cdot k_{d}^{j-1}\right)}{J}-M_{\mathrm{bkg}}\right]$$

Correction factors are typically necessary depending on the application, and include linearity (*k*_*L*_), fading (*k*_*F*_), and beam quality (*k*_*Q*_) which are standard and defined elsewhere.^19^ Angular dependence (*k*_*θ*_) describes the difference in signal in a CT environment as the angle of the detector is changed from lying flat in the bore, and the irradiation geometry correction (*k*_*G*_) accounts for the difference between a static en‐face irradiation and an irradiation based on a source rotating around the face of the detector.^18^

The calibration procedure determines the value of *N*_*D,W*_, and thereby establishes the relationship between the OSLD signal and dose. A common calibration procedure used clinically is that offered by the vendor, through pre‐irradiated dosimeters which are provided with the OSLD reader.^15^, ^16^ This set of 15 dosimeters are irradiated by the vendor in an 80 kVp beam to five known dose levels, and a calibration coefficient as a function of dose is determined for all future reading sessions. However, this is not an ideal calibration for several reasons. First, irradiation of calibration dosimeters is left solely to the vendor. Second, by performing only one calibration, instability or fluctuations in the OSLD reader are ignored, and drift in the reader over time is not accounted for. Third, variations in OSLD sensitivity between dosimeter production batches are neglected. Fourth, the vendor‐supplied calibration defines the dosimeter response relative to solely an 80 kVp beam. One of the main challenges in performing dosimetry using OSLD in diagnostic imaging is that the response of the dosimeter is very sensitive to changes in beam quality.^20^, ^21^, ^22^ Calibrating the dosimeter with a higher or lower energy than that being used for experimental measurements can introduce large errors into dose measurement unless appropriate energy correction factors (*k*_*Q*_) are used. However, the vendor calibration protocol does not address differences in energy between different scan parameters. Therefore, an alternative approach to OSLD calibration for CT measurements is desired. One approach was recently implemented by Stepusin et al.,^12^ where the OSLD readings at each energy and depth were cross‐calibrated with ion chamber measurements at each location to determine appropriate beam quality correction factors. Nevertheless, a systematic evaluation of calibration techniques, and associated uncertainties, is necessary.

The purpose of this investigation was, first, to present three calibration protocols: the vendor calibration procedure, a calibration using a 120 kVp CT beam and ion chamber, and a calibration using a megavoltage beamline (in this study using a ^60^Co beam) as would be reasonable for diagnostic procedures conducted at radiotherapy facilities). Second, we evaluated and contrasted the accuracy of these three protocols, particularly the suitability of the vendor calibration procedure. Third, we rigorously established the uncertainty associated with each protocol. To facilitate these three steps, we performed a range of CT dose measurements in different scans and at different locations, and assessed the dose determined with each of the three protocols. These doses were compared to the dose determined at these same locations with a calibrated ion chamber to validate the accuracy of the three different calibration approaches.

## 2 Calibration formalism

### 2.A Dosimeters

This work used the nanoDot OSL dosimeters and the InLight microStar OSL reader (Landauer, Inc., Glenwood, IL, USA). The aluminum oxide based dosimeter (Al_2_O_3_:C) has a disk‐shaped active volume 4 mm in diameter and 0.3 mm thick, and is enclosed in a light‐tight plastic cassette measuring 10 mm × 10 mm × 2 mm. The inLight microStar OSL reader was operated in continuous mode for a 7‐s read time, using the strong LED setting (for low doses). For this read time and LED intensity, the depletion was approximately 1.6% per reading,^18^ and this correction was applied to all subsequent readings. The relative element sensitivity was determined for each dosimeter by irradiating them all to 25 cGy and measuring their relative signals. These dosimeters were then bleached and used for the experiments described below. The derived element sensitivity correction factors were applied to all measurements.

Absolute dose in CT measurements was also measured with a RadCal (Monrovia, CA, USA) 10 × 5^−0.6^ CT ion chamber (farmer chamber style) with an active volume of 0.6 cm^3^. The chamber was previously calibrated in a 120 kVp beam by an accredited dose calibration lab (ADCL). The farmer‐type ion chamber was used in conjunction with a RadCal 9010 series electrometer, operated in autoexposure mode. To convert the measured exposure readings to absorbed dose using the ion chamber, we used the American Association of Physicists in Medicine (AAPM) task group (TG) report 111 protocol^23^ [eq. (3)] using the chamber reading (*q*), the calibration coefficient (*N*_*k*_), and appropriate corrections for temperature and pressure effects (*P*_*TP*_) and the electrometer factor (*P*_*elec*_). The ratio of mass energy absorption coefficients was also applied to define the dose to the medium of choice; for this work the medium selected was water.

(3) $$D_{\mathrm{water}}=k_{\mathrm{air}}{\left[\frac{{\overset{¯}{\mu }}_{\mathrm{en}}}{\rho }\right]}_{\mathrm{air}}^{\mathrm{water}}=qP_{\mathrm{TP}}P_{\mathrm{elec}}N_{k}{\left[\frac{{\overset{¯}{\mu }}_{\mathrm{en}}}{\rho }\right]}_{\mathrm{air}}^{\mathrm{water}}$$

### 2.B Calibration protocols

Optically stimulated luminescent dosimeter calibration establishes the relationship between dosimeter signal and dose using “standards” (i.e., OSLD irradiated to a known dose). For each of the three calibration procedures investigated, the OSLD calibration coefficient was defined using these “standards” as the ratio of the known dose to the OSLD signal (measured in counts) as shown in eq. (4).

(4) $$N_{D,W}=\frac{Delivered\;Dose\;(mGy)}{OSLD\;signal\;(counts)}$$

Water was selected as the reference medium for defining dose to the standard dosimeters using both the CT‐based calibration and the megavoltage‐based calibration. As a result, the absorbed dose calculated using the dosimeters and these protocols is dose to water, regardless of the actual measurement medium.

The process to determine this calibration coefficient for each of the three calibration protocols is described below:

#### 2.B.1 Vendor calibration protocol

This protocol used pre‐irradiated dosimeters provided by the dosimeter vendor. Calibration dosimeters are irradiated by the manufacturer on a PMMA phantom using 80 kVp x rays (2.9 mm Al HVL) to known delivered dose levels of approximately 0, 3, and 20 mGy. The two‐sigma uncertainty on the dose delivered to the dosimeters is reported to be ±5%.^24^

In this work, the calibration dosimeters were read three times each, and the average of the depletion‐corrected signal was used to establish the calibration factor: *N*_*D,Vendor*_. An adjustment for the difference in sensitivity in the vendor‐supplied dosimeters and the experimental dosimeters was also made (as these dots came from different production batches), despite the fact that correcting for this difference is not explicitly advised in the vendor calibration procedures: the calibration dosimeters had a mean inherent sensitivity of 0.85 while the experimental dots had a mean inherent sensitivity of 0.93. A uniform factor of the ratio of these (1.094) was therefore applied to the calibration factor to account for this difference.

#### 2.B.2 Free‐in‐air CT calibration protocol

This calibration protocol relates the dose measured using a calibrated ion chamber to the OSLD signal for dosimeters irradiated under identical conditions using a CT scanner as the radiation source.

**Figure 1:**
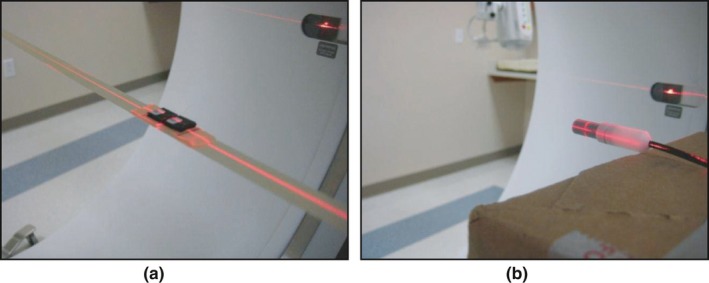
(a) OSLD setup for free‐in‐air CT calibration; (b) ion chamber setup using empty cardboard box for free‐in‐air calibration

In this work, the ion chamber and OSLD standards were irradiated free‐in‐air using a 120kVp CT beam (Discovery CT750 HD, GE Healthcare; Milwaukee, WI, USA). All calibration measurements were performed with a rotating CT tube using the medium bowtie filter and 64 × 0.625 mm detector configuration to provide a ~40 mm beam width at isocenter. The beam at this location had a measured HVL of 6.54 mm Al and a mean spectral energy of 59.9 keV (based on previous Monte Carlo simulations).^18^ No table motion was allowed during irradiation. Two nanoDots were positioned at isocenter using a piece of tape suspended through the CT bore (although any minimally attenuating support system would work). The OSLD were located at the machine isocenter using laser alignment lights, and the 40 mm beam width completely covered both dosimeters [Fig. 1(a)]. A single axial rotation was used to deliver 140 mA with a 1‐s rotation time. This procedure was repeated two additional times with new dosimeters, such that six total dosimeters were irradiated in the same fashion. Each of the six OSLDs was read three times, and the average (depletion and element sensitivity‐corrected) signal was used to describe the dosimeter signal.

The calibrated ion chamber was used to measure dose under identical conditions and scanning technique. The ion chamber was positioned on an empty cardboard box and aligned such that the active volume intersected the machine isocenter using laser alignment lights, and with the entirety of the chamber's active volume covered by the 40 mm beam [Fig. 1(b)]. This procedure was repeated two additional times to acquire three readings using the ion chamber.

The delivered dose (to water) was defined using the average of the ion chamber measurements [described in eq. (3)]. Per eq. (4), the calibration factor for the CT calibration (*N*_*D,CT*_) was the ratio of dose to signal.

#### 2.B.3 Calibration using megavoltage beam

The third calibration protocol used a ^60^Co beamline to determine dose (although a 6 MV beam could also be used), and followed the general procedure used for OSLD calibration by IROC Houston and other radiotherapy auditing bodies.^3^, ^5^, ^6^ This procedure relies on megavoltage equipment, and would therefore be most applicable to radiotherapy environments. One advantage of this protocol is the very high accuracy of the delivered dose to the calibration (standard) OSLDs by virtue of the high precision and stability in the clinical reference dosimetry of megavoltage beams.

**Figure 2:**
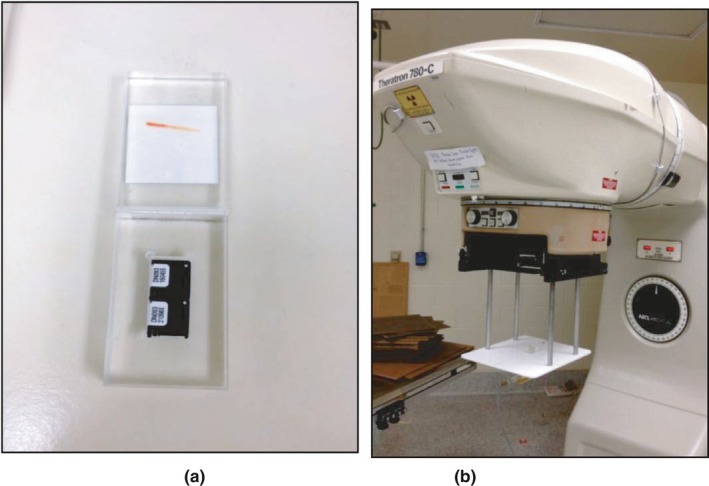
(a) Acrylic block with 2 OSLD used for irradiating OSLD standards in ^60^Co Calibration Protocol (b) ^60^Co unit used for irradiation of standards in the acrylic block.

In this work, irradiations were performed using a Theratron 780C cobalt unit (AECL/Theratronics International Ltd., Kanata, ON, Canada), which is maintained by an ADCL at The MD Anderson Cancer Center (Houston, TX, USA). Two dosimeters were simultaneously irradiated in a Lucite block approximately 4 cm × 4 cm × 4 cm in dimension [Fig. 2(a)], positioned in a jig to minimize setup uncertainty [Fig. 2(b)], and located at a distance of 80 cm from the cobalt source. The beam energy at the in‐air location of the calibration measurements was taken as 1.25 MeV.

The dose delivered to the dosimeters (45 mGy) was determined based on a decay‐correction of the dose rate of the source and the TG‐51 calibration protocol.^25^

Four dosimeters were irradiated using the ^60^Co unit. Each of the four dosimeters was read three times, and the average (depletion and element sensitivity‐corrected) signal was used to describe the dosimeter signal. This OSLD signal was compared to the delivered dose with the cobalt beam based on the timer setting and the calculated dose rate. This yielded the calibration coefficient for the ^60^Co calibration protocol: *N*_*D,60Co*._

### 2.C Correction factors (*k*_*i*_)

Because the conditions of these calibration irradiations will not be identical to the conditions of the irradiation of experimental OSLD, correction factors to the measured signal are necessary. The necessary correction factors for each of the above calibration protocols are described below in the context of the test measurements used to evaluate the accuracy of the different calibration procedures.

### 2.D Test measurements

A set of measurements was performed to compare the results of the three calibration protocols with the dose measured using a RadCal 10 × 5 CT farmer‐type ion chamber. All measurements were performed using a 64‐slice CT scanner (Discovery CT750 HD, GE Healthcare, Milwaukee, WI, USA) and standard acrylic CT Dose Index (CTDI) phantoms (32 and 16 cm in diameter). The half value layer of the 80, 120, and 140 kVp beams (with medium bowtie filter) were 4.27, 6.54, and 7.62 mm Al, respectively. The OSLD were placed in the center, the periphery, and on the surface of the large CTDI phantom and in the center and on the surface of the small CTDI phantom. For each measurement location, two OSLD were irradiated, and the irradiation was repeated three times for a total of six dosimeters at each location. The irradiation was then repeated with the farmer‐type ion chamber in the same location as the OSLD. The OSLD were read three times and the average (depletion and element sensitivity‐corrected) value was used as the corrected OSLD signal. This same signal was converted to dose using the three different calibration protocols described above, so any differences in measured dose for a given condition reflect differences in the calibration protocol (including associated correction factors).

The CT‐scanning techniques used to compare the calibration protocols were selected to represent a range of energy spectra. Eleven unique measurement conditions were used for this comparison; the scanning parameters and dosimeter position for these 11 cases are shown in Table 1. All scans used a 40 mm beamwidth and either axial or helical scans, depending on scan extent (as shown in Table 1). All helical scans used a pitch of 0.984. Exposures ranged from 200 to 900 mAs with either 1 or 2 s rotations times, such that the dose delivered to the ion chamber at the same position was between 25 and 40 mGy. Each of these conditions was also simulated using a previously benchmarked Monte Carlo model based on the same x‐ray source and geometry^26^; the mean spectral energy at the position of the dosimeter was determined (Table 1).

**Table 1:** Measurement conditions for calibration protocol validation measurements

Measurement position	CTDI phantom	Position	kVp	Scan extent (mm)	Scan Type	Mean spectral energy (keV)
1	16 cm	Center	80	40	Axial	45.4
2	16 cm	Surface	80	150	Helical	47.9
3	32 cm	Center	120	150	Helical	51.7
4	32 cm	Center	120	40	Axial	54.7
5	16 cm	Center	120	40	Axial	55.3
6	16 cm	Center	140	40	Axial	58.1
7	32 cm	Center	140	40	Axial	58.7
8	32 cm	Periphery	120	40	Axial	59.5
9	32 cm	Surface	120	150	Helical	60.7
10	16 cm	Surface	140	150	Helical	63.2
11	16 cm	Surface	140	40	Axial	64.9

The dose to the OSLD was determined using eqs. (1) and (2). For most uses in a CT environment, *k*_*L*_ (linearity correction) is unity.^18^ All dosimeters were placed flat relative to the CT bore so that there was no angular dependence, and all times were controlled so there was no relative fading between the standards and experimental detectors. Other possible corrections are described below:

For the vendor‐recommended calibration procedure, the dose was determined using the manufacturer‐provided approach, as shown in eq. (5), and using the energy correction provided by the vendor (*k*_*Q, Vendor*_), which the vendor states is equal to 1.19 for all CT measurements. Other possible correction factors were ignored as no other corrections are suggested by the vendor.

(5) $$D_{\mathrm{Vendor}}={\bar{M}}_{\mathrm{corr}}\times N_{D,Vendor}\times k_{Q,Vendor}$$

For CT calibration and dosimetry, the value of *k*_*G*_ is unity because there is no difference in irradiation geometry between the standards and experimental OSLD. The dose to the OSLD was therefore calculated using eq. (6).

(6) $$D_{\mathrm{CT}}={\bar{M}}_{\mathrm{corr}}\times N_{D,CT}\times k_{Q,CT}$$

Values for *k*_*Q,CT*_ were previously determined using the simulated photon energy spectra and Burlin cavity theory,^18^ and are provided in Table 2.

**Table 2:** Energy correction factor values for two different calibration protocols

Measurement position	kVp	Mean spectral energy (keV)	*k*_*Q,CT*_	*k*_*Q,Co‐60*_
1	80	45.4	0.82	0.29
2	80	47.9	0.85	0.30
3	120	51.7	0.88	0.31
4	120	54.7	0.91	0.33
5	120	55.3	0.93	0.33
6	140	58.1	0.97	0.35
7	140	58.7	0.96	0.34
8	120	59.5	1.00	0.36
9	120	60.7	1.02	0.36
10	140	63.2	1.06	0.38
11	140	64.9	1.09	0.39

For the megavoltage calibration technique, *k*_*G*_ is necessary to account for the differences in irradiation geometry between the calibration conditions (static en‐face irradiation of the dosimeter) and the measurement conditions (rotating irradiation), but has a relatively small value of 1.03.^18^ The dose to the OSLD for dosimeters following the ^60^Co calibration protocol was determined using eq. (7), where the energy correction factors are shown Table 2.

(7) $$D_{60_{\mathrm{Co}}}={\bar{M}}_{\mathrm{corr}}\times N_{D,60_{\mathrm{Co}}}\times k_{G}\times k_{Q,60_{\mathrm{Co}}}$$

### 2.E Uncertainty analysis

Although eqs. ((5), (6), (7)) are of similar form, the components of each are derived from different sources or have a different impact on the total uncertainty and therefore each calibration protocol has a different overall uncertainty. The total uncertainty of the determined dose was therefore calculated for each calibration protocol. This was based on the uncertainty in the underlying factors: the uncertainty in the factors *k*_*L*_, *k*_*F*_, *k*_*G*_, *k*_*θ*_, and *k*_*Q*_ were taken from Scarboro et al.^18^ (except for the uncertainty in the vendor‐recommended CT energy correction factor, which was estimated based on the actual measured energy correction factors for the range of measurement conditions investigated). The variance in the raw count rate was taken from the measurements in the current study. The variance in each *N*_*D*_ was determined from the uncertainty in each of its parameters (i.e., the parameters in eq. (1) rearranged to solve for *N*_*D*_), including the uncertainty in the reference dose associated with each calibration protocol (±5% for the vendor calibration,^24^ ±5% for the CT protocol,^27^ and 0.9% for the MV protocol^28^).

Rather than simply combining these component uncertainties in quadrature, the total uncertainty was determined more robustly using eqs. (8) and (9), where *var* denotes variance and *E* denotes the expected value (i.e., the mean).

(8) $$var\left(XY\right)=var\left(X\right)var\left(Y\right)+var\left(X\right)E{\left(Y\right)}^{2}+var\left(Y\right)E{\left(X\right)}^{2}$$

In eq. (8), *X* represented a given factor (e.g., ${\bar{M}}_{\mathrm{corr}}$), and Y represented the product of the remaining factors (*N*_*D*_ *× k*_*L*_ *× k*_*F*_ *× k*_*G*_ *× k*_*θ*_ *× k*_*Q*_). The variance of Y was calculated by applying eq. (8) recursively to each individual factor (X’ = *N*_*D*_, and Y’ =* k*_*L*_ *× k*_*F*_ *× k*_*G*_ *× k*_*θ*_ *× k*_*Q*_ and so on). Calculation of the variance of ${\bar{M}}_{\mathrm{corr}}$ also required eq. (9), as ${\bar{M}}_{\mathrm{corr}}$ is a linear combination of products of correlated variables [eq. (2)]. In this case, CoV is the covariance between X and Y:

(9) var(X+Y)=var(X)+var(Y)+2CoV(X,Y)

This method is more robust than adding uncertainties in quadrature because it does not rely on the normality assumption of the distribution, or on any type of mathematical approximation, and allowed us to analytically determine the standard deviation of the OSLD measured dose.

## 3 Results and discussion

### 3.A Comparison of calibration methods

**Figure 3:**
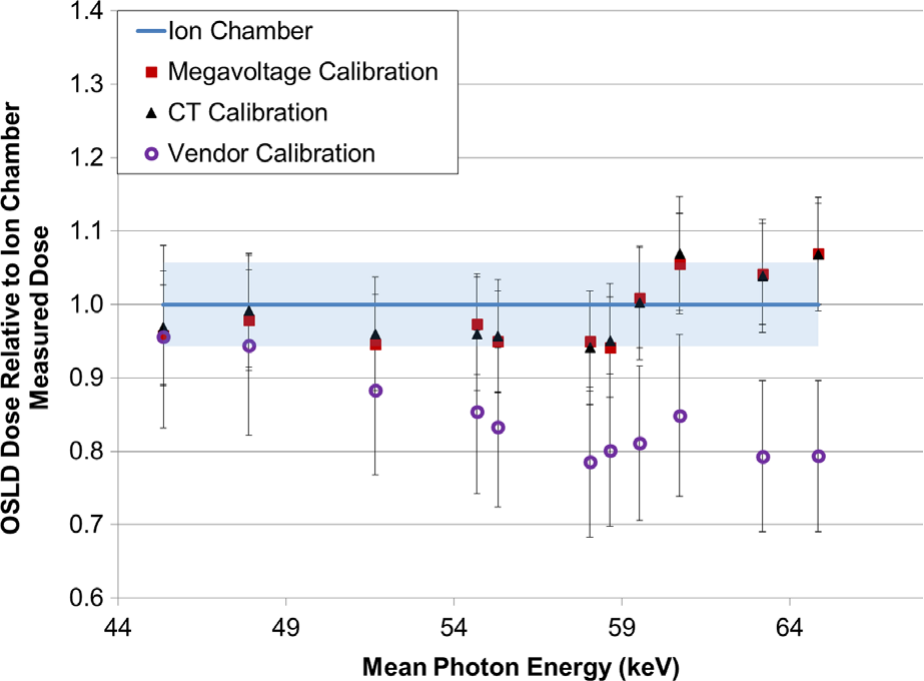
Comparison of three OSLD measured doses (using different calibration protocols) to ion chamber measured dose. Error bars on each dataset represent the total uncertainty for each measurement. The shaded region represents the uncertainty associated with the ion chamber reading.

For a range of measurement conditions, varying measurement position, phantom size, kVp, and scan extent, the dose to the OSLD was determined using each of three calibration protocols. These values were then compared to the dose measured using the farmer‐type CT ion chamber and plotted as a function of the mean photon energy for the scan parameters selected (Fig. 3).

The error bars in Fig. 3 represent the relative uncertainty for each method, as calculated in eqs. (8) and (9). A comparison of the measured doses is provided in Table 3, along with the percent difference from the ion chamber measured dose.

**Table 3:** For 11 different measurement locations, the dose determined with a farmer‐type ion chamber is presented. This is compared to the dose measured with OSLD using three independent calibration protocols

Measurement condition	kVp	Mean spectral energy (keV)	Ion chamber	Vendor calibration		In‐air CT calibration		^60^Co calibration	
			Dose (mGy)	Dose (mGy)	% diff	Dose (mGy)	% diff	Dose (mGy)	% diff
1	80	45.4	31.8	30.4	4.4	30.8	3.2	30.5	4.2
2	80	47.9	28.1	26.6	5.6	27.9	0.8	27.5	2.1
3	120	51.7	29.8	26.3	11.7	28.6	4.0	28.2	5.4
4	120	54.7	28.7	24.5	14.6	27.5	4.0	27.9	2.7
5	120	55.3	34.1	28.4	16.7	32.6	4.3	32.3	5.1
6	140	58.1	33.7	26.5	21.5	31.7	5.9	32.0	5.0
7	140	58.7	34.2	27.4	19.9	32.5	4.9	32.2	5.9
8	120	59.5	36.3	29.4	18.9	36.4	0.2	36.6	0.9
9	120	60.7	27.1	23.0	15.2	29.0	6.9	28.6	5.5
10	140	63.2	34.1	27.1	20.7	35.4	3.9	35.5	4.1
11	140	64.9	32.1	25.5	20.7	34.3	6.8	34.3	6.9
Average					15.5		4.1		4.4

The results of the calibration protocol comparison indicate that similar results can be expected using either the CT‐based or megavoltage‐based calibration method. On average, the absolute difference between the dose determined using OSLD and one of these two methods versus that determined directly using a CT ion chamber was less than 5%.

The vendor‐recommended calibration shows good agreement with the ion chamber for very low experimental energies (80 kVp). However, as the CT energy increases, the agreement between the OSLD and the ion chamber deteriorates—exceeding 20% disagreement for many scan techniques. Although the vendor energy correction factor (1.19) was applied it does not appear adequate for the range of energies seen in clinically relevant scans. For the set of conditions examined in this study, the vendor‐recommended calibration technique underestimated the dose by an average of 15.5%. This result is consistent with the recent work by Vrieze et al.,^29^ who used the manufacturer‐recommended calibration procedure and found that the OSLD systematically under‐responded relative to ion chamber readings.

Both the CT‐based calibration and the megavoltage‐based calibration have been shown to be strong alternatives to the vendor‐provided approach for calibrating OSLD nanoDots. Each of these two methods provides a measure of dose well within 10% of a CT ion chamber for a wide range of scan conditions. For the scan conditions examined, a very specific energy correction factor was applied to account for variations in the spectra between the calibration conditions and the experimental conditions. Such precise spectral information is rarely known for a particular measurement condition, and energy correction factors based on more general scan parameters (kVp, phantom size, scan extent, etc.) are more useful for clinical application. The sensitivity of *k*_*Q*_ to different scan parameters was previously found to be most sensitive to kVp and measurement location, and to be relatively insensitive to scan extent or phantom/patient size (usually less than a 2% effect).^18^ Consequently, values for *k*_*Q*_ can be well approximated based solely on nominal kVp and measurement position with only a small increase in measurement uncertainty. To determine recommended values for *k*_*Q*_ for the CT‐based calibration protocol, we averaged previously determined values of *k*_*Q*_ across different phantom sizes and scan extents.^18^ This provided average *k*_*Q*_ values based solely on kVp and measurement position (Table 4).

**Table 4:** Values of energy correction factor *k*_*Q*_ relative to the CT‐based calibration protocol for a range of CT parameters

kVp	Surface	Periphery (1 cm depth)	Center
80	0.85	0.83	0.81
120	1.03	0.98	0.90
140	1.10	1.03	0.94

For a calibration relative to a megavoltage beam, the value of *k*_*Q*_ is far from unity because of the large difference in energy between calibration and measurement. Table 5 contains the recommended values for *k*_*Q*_ for this protocol based on the same pooling of previously determined *k*_*Q*_ values^18^ based solely on kVp and measurement position. While these values were calculated specifically for ^60^Co, they could also be used for 6 MV beams within 2%.^7^, ^20^

**Table 5:** Values of energy correction factor *k*_*Q*_ relative to the ^60^Co based calibration protocol for a range of CT parameters

kVp	Surface	Periphery (1 cm depth)	Center
80	0.30	0.30	0.29
120	0.37	0.35	0.33
140	0.39	0.37	0.34

The values provided in Tables 4 and 5 allow for simple and accurate energy correction using either of the two proposed calibration protocols.

Because the spectra may vary between different scanner manufacturers, these values should be applied with caution. While many CT scanners have similar spectra, Toshiba scanners have been known to have a softer spectrum, and therefore will have a different *k*_*Q*_ correction factor relative to a distinct calibration beam (i.e., the vendor's calibration beam or a megavoltage beam; e.g., *k*_*Q*_ in Table 5). A strength of the CT calibration procedure is that the calibration and experimental readings can be done on the same CT scanner. If the CT spectrum is inherently softer than evaluated in this study, this will be substantially accounted for in the calibration procedure and thereby mitigate the effect of a different calibration CT spectrum. While this will help mitigate substantial uncertainty in *k*_*Q*_ from mapping between, for example, a ^60^Co beam and an arbitrary CT calibration beam (e.g., Table 5), the relative variations in the spectrum of any CT beam (i.e., different beam hardening and scattering between the calibration and experimental dosimeters as shown in Table 4) will nevertheless persist. The magnitude of these differences may be similar to the values shown here in Table 4, but they will not be identical.

### 3.B Uncertainty analysis

There are both random and systematic uncertainties associated with the determination of dose using this OSLD system. The uncertainties arise from measurement imprecision in the OSLD signal as well as uncertainties in the various correction factors applied to the signal.

When averaged over the three readings, and as detailed in Table 6, the relative uncertainty in the corrected OSLD signal was consistent regardless of the calibration protocol: 1.3%. This originated with the relative uncertainty in the depletion‐corrected raw OSLD reading (0.8%) and the relative uncertainty in the element specific sensitivity factor (1.0%). The uncertainty in *N*_*D*_ was dominated by the uncertainty in the delivered dose to the standard dosimeters, which was lowest for the megavoltage calibration protocol and produced an uncertainty in *N*_*D,60Co*_ of 1.6%. In contrast, the uncertainty in the calibration coefficient using the other two protocols had a value of slightly over 5%. Finally, the total uncertainty in the dose determination using each protocol included the uncertainty in the correction factors. The largest component of uncertainty in this step was from the beam quality correction factors. The relative uncertainty in the corrected OSLD reading, the calibration coefficient, and the overall dose determination for the three calibration protocols is shown in Table 6.

**Table 6:** Relative uncertainties (at the 2‐sigma level) in corrected OSLD signal, calibration coefficient, and dose determination for each calibration protocol

	Vendor calibration	CT free‐in‐air calibration	^60^Co calibration
Uncertainty in OSLD reading $\frac{\sigma_{{\bar{M}}_{\mathrm{corr}}}}{{\bar{M}}_{\mathrm{corr}}}$	±1.3%	±1.3%	±1.3%
Uncertainty in calibration coefficient $\frac{\sigma_{N_{D,W}}}{N_{D,W}}$	±5.2%	±5.3%	±1.6%
Total uncertainty in calculated dose $\frac{\sigma_{D}}{D}$	±13.5%	±8.3%	±8.5%

The largest overall uncertainty was associated with the vendor calibration, largely because of the simplistic management of the energy dependence of the detector. When comparing the CT and the ^60^Co‐based protocols, the ^60^Co protocol had better precision in the calibration coefficient, but because it had much larger correction factors, it had a larger uncertainty associated with these correction factors, leading to a slightly larger overall uncertainty.

The calibration protocol with the lowest overall uncertainty was the CT‐based calibration, and this is likely the best calibration option for most diagnostic CT clinics. The relative uncertainty in dose measurement using properly calibrated OSLD is ±8.3% (2‐sigma), which is reasonable for CT dosimetry applications. Precise dosimetry is also achievable using a megavoltage calibration, and this approach may be of interest to radiotherapy clinics due to their familiarity with megavoltage equipment. The relative uncertainty on the vendor calibration protocol was the highest of the three examined. This is largely due to the uncertainty in dose delivered to standards, as well as uncertainty in the correction factor to account for energy or other effects. However, the vendor calibration is also sensitive to systematic uncertainties; for example, as discussed above, the results will be systematically different for a CT scanner with a different energy spectrum.

## 4 Conclusion

In this work, two calibration protocols are presented which are strong alternatives to the vendor‐supplied calibration method for performing CT dosimetry using the nanoDot OSLD. The CT free‐in‐air calibration requires a previously calibrated ion chamber, and OSLD standards to be irradiated with a consistent and reproducible scan technique. Energy correction factors are generally necessary using this calibration technique and a simple table of factors is provided that cover a wide range of scan conditions. The megavoltage calibration requires a larger correction factor, but offers comparable accuracy. Using either the CT free‐in‐air or megavoltage‐based calibration approaches, point dosimetry with a relative uncertainty of less than ±10% is readily achievable in a CT environment.

## Conflict of interest

No conflicts of interest.
